# Giant Mucinous Borderline Ovarian Tumor With Microinvasion Presenting As Massive Ascites and Acute Respiratory Compromise: A Case Report

**DOI:** 10.7759/cureus.89504

**Published:** 2025-08-06

**Authors:** Stefanos Flindris, Effrosyni Styliara, Georgia Galaziou, Stamatios Petousis, Chrysoula Margioula-Siarkou, Minas Paschopoulos, Iordanis Navrozoglou

**Affiliations:** 1 Second Department of Obstetrics and Gynecology, School of Medicine, Aristotle University, Thessaloniki, GRC; 2 Department of Radiology, University Hospital of Ioannina, Ioannina, GRC; 3 Department of Obstetrics and Gynecology, University Hospital of Ioannina, Ioannina, GRC; 4 Department of Obstetrics and Gynecology, University of Ioannina, Ioannina, GRC

**Keywords:** controlled cyst decompression, giant ovarian tumor, midline laparotomy, mucinous borderline neoplasm, respiratory compromise

## Abstract

A 34-year-old nulligravida with schizophrenia presented after four months of progressive abdominal distension, culminating in severe respiratory compromise. An urgent transabdominal ultrasound was initially interpreted as massive ascites; however, its diagnostic accuracy was limited by the extreme abdominal distension and the patient’s inability to change position due to respiratory distress. Further evaluation with CT of the thorax and abdomen revealed a 35 × 42 × 48.5 cm cystic mass arising from the left ovary, causing marked thoracoabdominal compression. Laboratory studies demonstrated leukocytosis, elevated inflammatory markers, and increased carcinoembryonic antigen and carbohydrate antigen 19-9, consistent with an ovarian neoplasm. An urgent midline laparotomy permitted controlled decompression of 37 L of serosanguinous fluid and en bloc resection of the ovarian mass, relieving the patient’s respiratory distress. Histopathology identified a mucinous borderline tumor with focal intraepithelial carcinoma, microinvasion (<5 mm), and an incidental benign Brenner component. After an uneventful recovery, the patient elected definitive management and underwent total hysterectomy with right salpingectomy one month later. At six-month follow-up, she remained in good health without evidence of recurrence.

## Introduction

Ovarian mucinous neoplasms comprise a spectrum of epithelial tumors defined by multiloculated cysts filled with abundant intracellular mucin. Their lining epithelium is predominantly of the gastrointestinal type, most commonly gastric foveolar or enteric mucinous cells, with Müllerian (endocervical) differentiation observed only rarely [1]. According to the World Health Organization classification, about 80% present as benign mucinous cystadenomas, roughly 10% as borderline (atypical proliferative) tumors, and the remaining 10% as invasive carcinomas, with the latter frequently representing secondary involvement from gastrointestinal or pancreatic primaries [2].

Borderline mucinous tumors are distinguished by epithelial stratification, papillary architecture, and increased mitotic figures without destructive stromal invasion. Microinvasion, defined as stromal infiltration under 5 mm, occurs in 3-5% of borderline cases and confers a modestly higher risk of recurrence, although complete surgical excision yields excellent long-term outcomes [3]. Unilateral salpingo-oophorectomy remains the cornerstone of treatment for both benign and borderline lesions. Immunohistochemical profiling (CK7, CK20, hormone receptors, proliferation indices) further refines prognostic assessment and confirms gastrointestinal-type differentiation [3].

Despite advances in imaging and routine pelvic evaluation, a subset of women, often those with nonspecific symptoms or barriers to care, present late with tumors exceeding 10 cm in diameter. Such “giant” masses may precipitate life-threatening complications, including respiratory compromise from diaphragmatic elevation, hemodynamic instability due to caval compression, and intestinal obstruction [4]. The sudden hemodynamic shifts that follow rapid tumor decompression at surgery heighten the risk of pulmonary edema, mandating meticulous preoperative cardiovascular and respiratory optimization [4,5].

Initial evaluation of an adnexal mass begins with a detailed ultrasound assessment using standardized morphological criteria as well as risk scoring systems such as the Risk of Malignancy Index or IOTA models, together with serum cancer antigen 125 (CA 125) measurement to estimate malignancy risk. In cases of extremely large ovarian cysts, however, these tools may prove misleading because massive fluid collections can resemble ascites and confound imaging interpretation. As a result, the decision to proceed by laparoscopy or open laparotomy must be made based on each patient’s age, menopausal status, comorbidities, and the lesion’s size and imaging characteristics [6]. Tait and Miller reported a 156-pound ovarian tumor, which is a paradigm that underscores both the technical obstacles presented by giant neoplasms and the imperative of a multidisciplinary team to secure a safe and complete resection [7].

In this report, we describe the case of a young woman with a giant mucinous borderline ovarian tumor harboring microinvasive foci, whose massive cystic mass masqueraded as ascites and precipitated acute respiratory distress, underscoring both the diagnostic challenges and the operative complexities of managing such extensive neoplasms.

## Case presentation

A 34-year-old G0P0 woman, with a normal menstrual history and no prior abdominal surgeries, was referred to the emergency department of the University Hospital of Ioannina with four months of progressive abdominal distention and mild generalized abdominal pain. Her medical history was significant for schizophrenia diagnosed in early adulthood, managed with lorazepam 2.5 mg twice daily and amisulpride 400 mg twice daily. She had no history of human papillomavirus vaccination, tobacco use, or known allergies. Her family history was notable for breast cancer in her grandmother and ovarian cancer in her aunt.

On examination, the abdomen exhibited massive distention without peritoneal signs. The lungs were compressed by the elevated diaphragm, and oxygen therapy at 3 L/minute via a nasal cannula provided insufficient relief. Laboratory tests revealed leukocytosis (white blood cell count of 13,600/µL, with 82% neutrophils), thrombocytosis (platelet count of 543,000/µL), elevated C-reactive protein (12 mg/dL), and thyroid-stimulating hormone (TSH) of 5.65 µIU/mL. Tumor markers demonstrated alpha-fetoprotein (AFP) of 3.4 ng/mL, carcinoembryonic antigen (CEA) of 1.2 ng/mL, carbohydrate antigen 19-9 (CA 19-9) of 639 U/mL, cancer antigen 15-3 (CA 15-3) of 17 U/mL, and CA 125 of 55 U/mL. Her blood pressure was 130/80 mmHg, pulse rate was 95 beats/minute, and respiratory rate was 21 breaths/minute.

**Figure 1:**
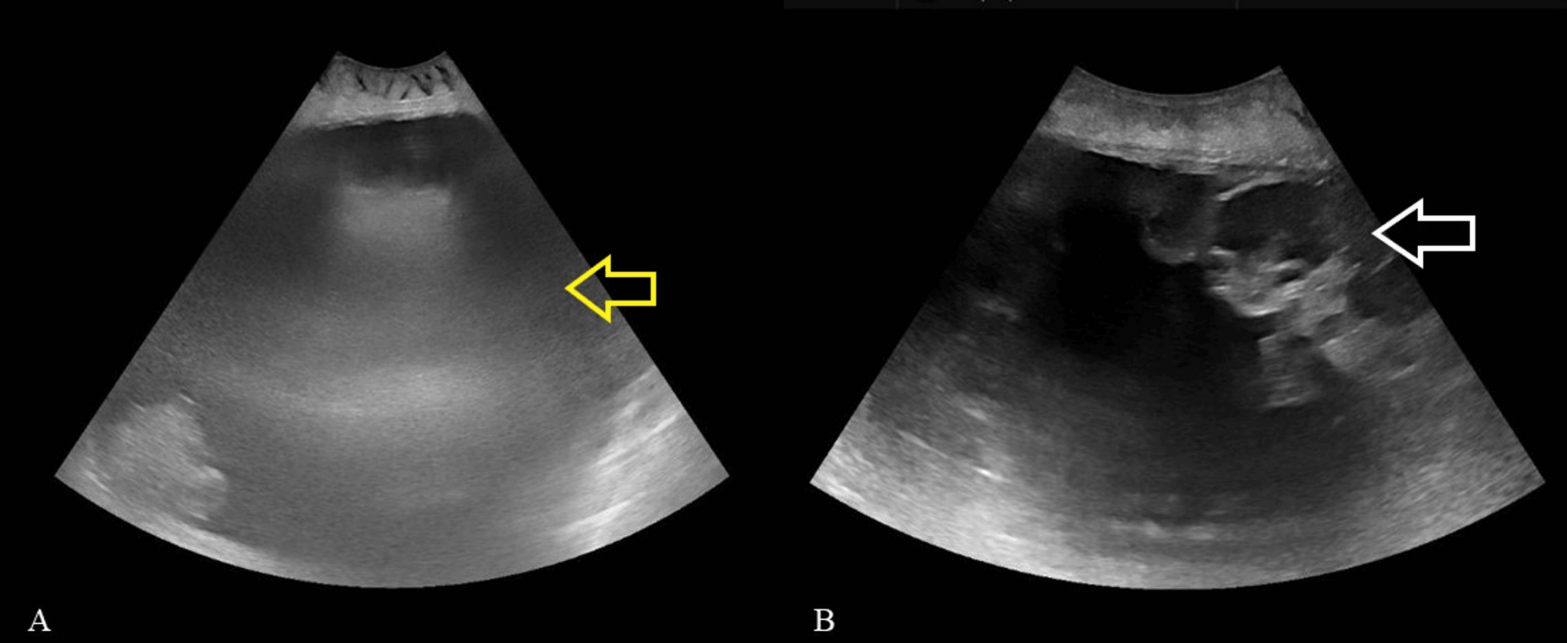
(A) Axial midline transabdominal ultrasound image demonstrating a massive fluid collection occupying nearly the entire peritoneal cavity (yellow arrow). (B) Axial transabdominal ultrasound image at the level of the lower abdomen demonstrating the massive anechoic fluid collection displacing adjacent small bowel loops laterally (white arrow).

**Figure 2:**
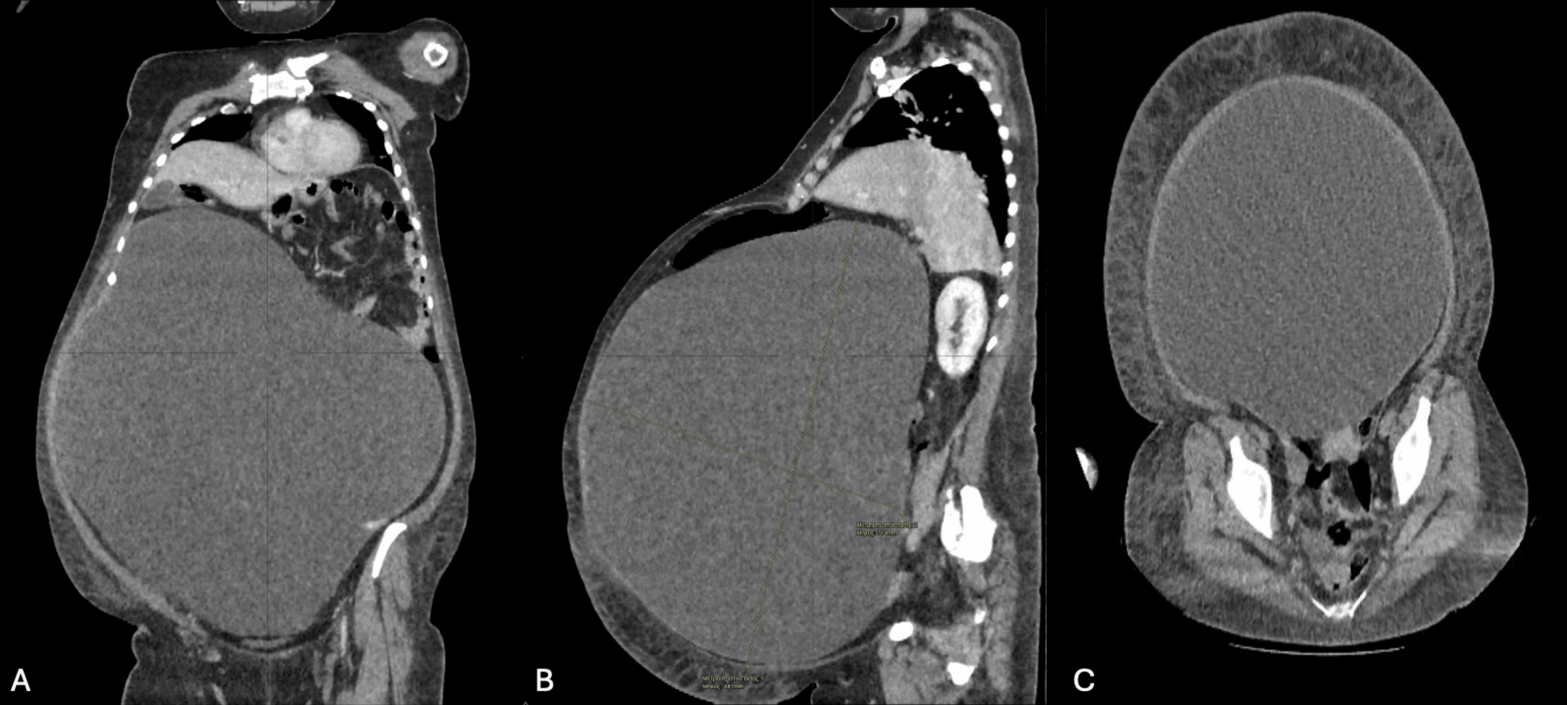
(A) Coronal CT: giant unilocular, low-attenuation cyst occupying most of the abdominal cavity and displacing bowel loops. (B) Sagittal CT: orthogonal calipers demonstrate the cyst’s maximal craniocaudal (44.1 mm) and anteroposterior (31.4 mm) dimensions. (C) Axial CT: homogeneous, thin-walled cystic lesion exerting mass effect on the pelvic organs and great vessels.

Abdominal ultrasound was technically challenging and of limited diagnostic utility due to the markedly distended abdomen. The extreme depth of the abdominal cavity hindered visualization of pelvic structures, rendering the uterus and ovaries indiscernible. Moreover, the assessment of fluid mobility was not possible because the patient’s respiratory distress made her unable to alter positions (Figures 1A, 1B). CT of the abdomen and thorax demonstrated a 30 × 40 × 45 cm predominantly cystic mass extending into the upper abdomen (mostly on the right), displacing bowel posteriorly, compressing the inferior vena cava and descending aorta, most likely arising from the left ovary. A separate 31 mm lesion adjacent to the uterus likely represented the right ovary (Figures 2A-2C).

**Figure 3:**
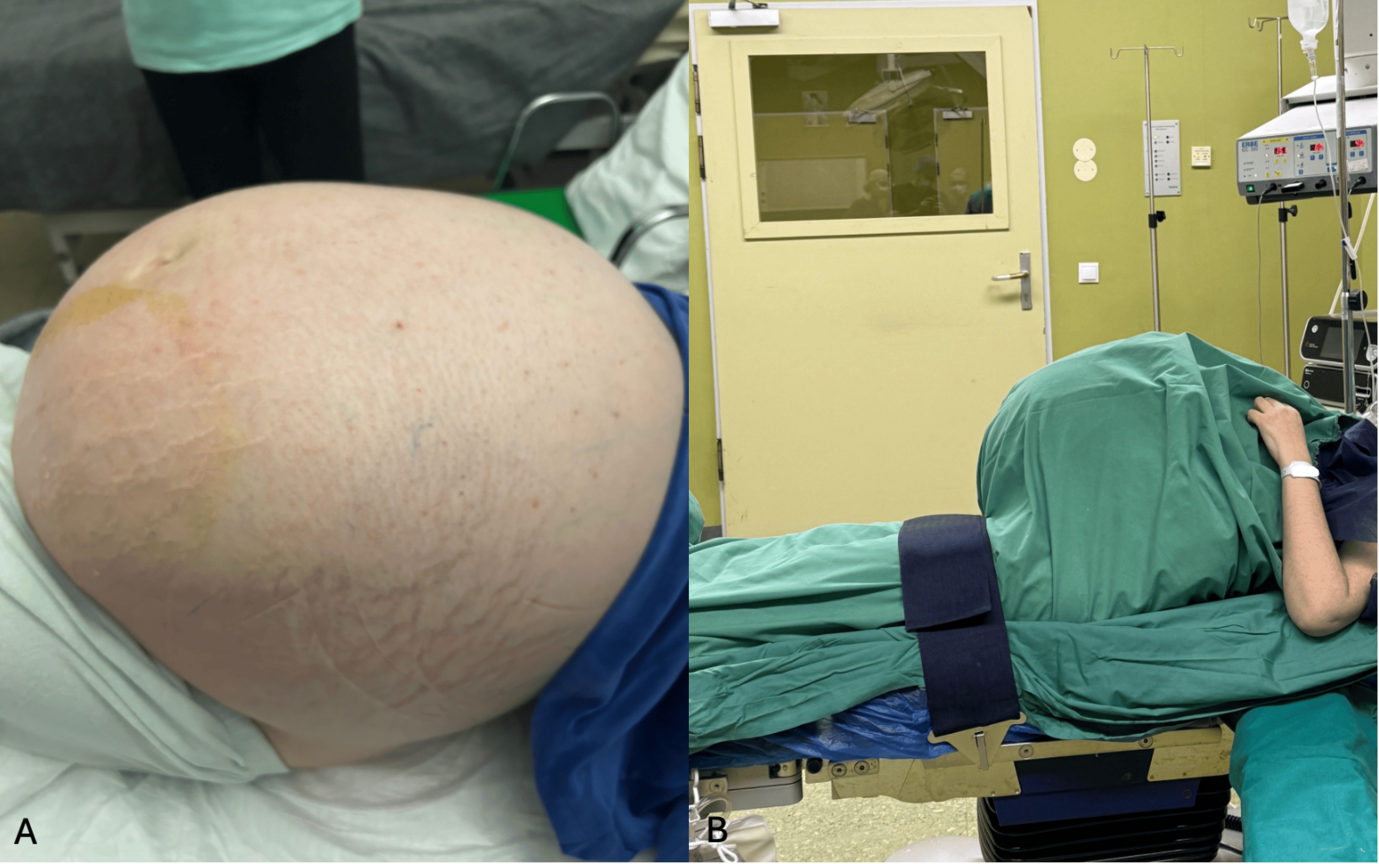
(A) Massive abdominal distension from a giant ovarian mass. (B) Intraoperative lateral profile highlighting the pronounced abdominal protuberance.

Due to progressive respiratory distress unresponsive to a 50% Venturi mask at 10 L/minute, arterial blood gas analysis showed a pH of 7.30, PaO₂ of 60 mmHg, PaCO₂ of 55 mmHg, HCO₃⁻ of 26 mEq/L, and SaO₂ of 90%. A summary of key laboratory findings on admission, including hematology, inflammatory markers, endocrine function, tumor markers, and arterial blood gas values, is presented in Table 1. An emergent exploratory laparotomy was performed under general anesthesia. Invasive hemodynamic monitoring, including an arterial line and central venous pressure catheter, guided fluid management to avoid both hypovolemia and fluid overload. As demonstrated in Figure 3A, the patient exhibited massive abdominal distension due to a giant ovarian mass, and the intraoperative lateral profile in Figure 3B highlights the pronounced abdominal protuberance. The patient was positioned with slight head elevation, and positive‐pressure ventilation was titrated to minimize atelectasis and optimize oxygenation.

**Table 1:** Summary of key laboratory parameters on admission, including hematology, inflammatory markers, endocrine function, tumor markers, and arterial blood gas values.

Test	Result	Units	Reference range
Hematology and inflammation			
White blood cell count	13.6	10³/µL	4.0–11.0
Neutrophils	82	%	40–70
Platelets	543	10³/µL	15–400
C-reactive protein	12.0	mg/dL	-
Endocrine			
Thyroid-stimulating hormone	5.65	µIU/mL	0.4–4.0
Tumor markers			
Alpha-fetoprotein	3.4	ng/mL	-
Carcinoembryonic antigen	1.2	ng/mL	-
Cancer antigen 19‑9	639	U/mL	-
Cancer antigen 15‑3	17	U/mL	-
Carbohydrate antigen 125	55	U/mL	-
Arterial blood gas			
pH	7.30	-	7.35–7.45
PaO₂	60	mmHg	75–100
PaCO₂	55	mmHg	35–45
HCO₃⁻	26	mEq/L	22–26
SaO₂	90	%	95–100

**Figure 4:**
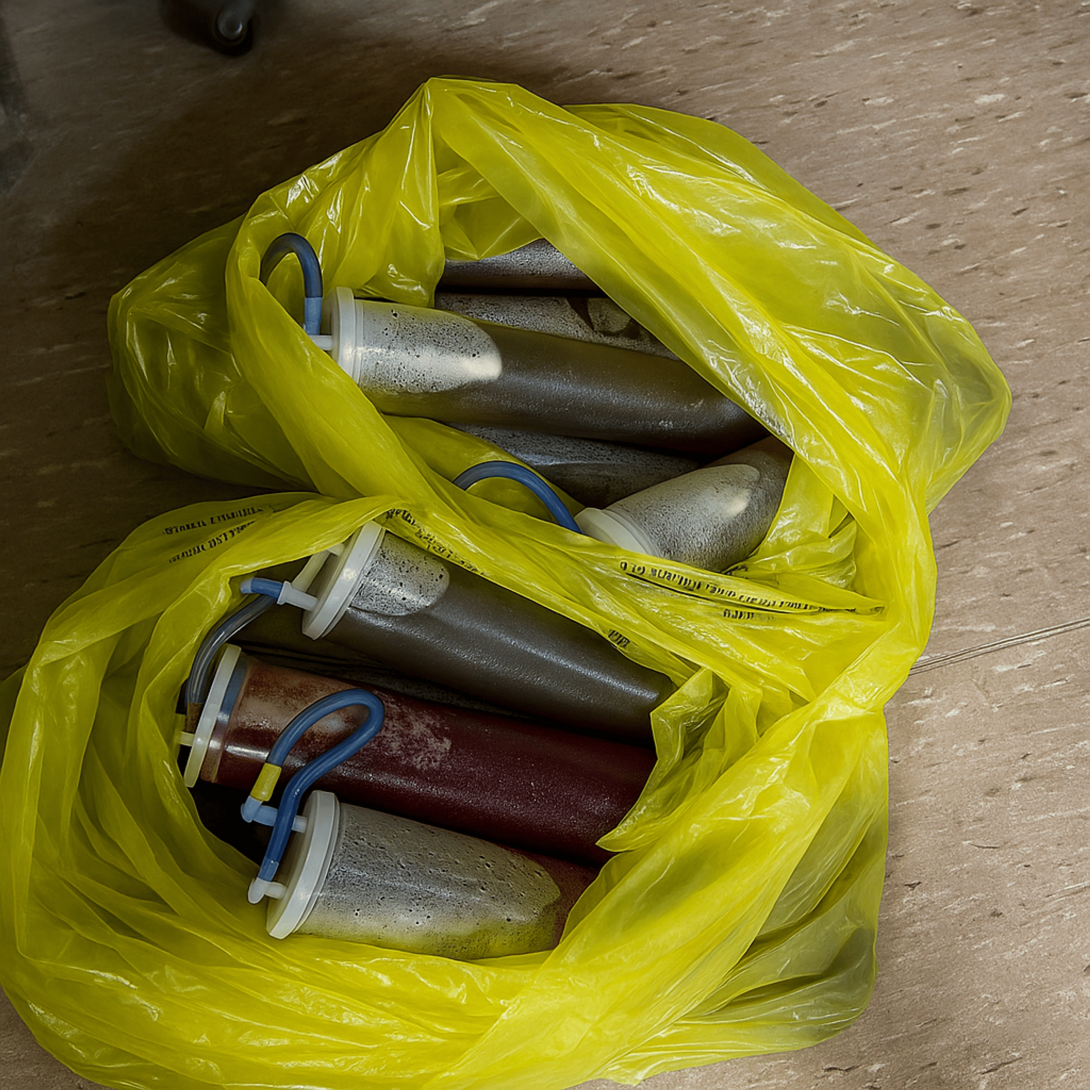
Aspirated ovarian cyst fluid collected in suction canisters.

**Figure 5:**
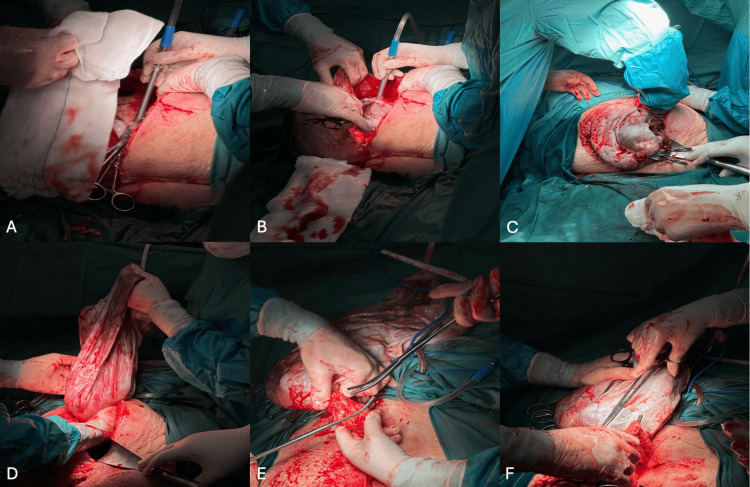
(A) Midline laparotomy revealing the ovarian capsule. (B) Initial dissection of the cyst wall. (C) Exteriorization of the mass through the wound. (D) Delivery of the ovarian mass with gentle traction. (E) Clamping and isolation of the infundibulopelvic ligament. (F) Final mobilization of the mass before resection.

A midline incision to the umbilicus exposed the ovarian cyst, which was carefully isolated with moistened gauze packs. Gradual decompression was achieved by making a small incision in the cyst wall and applying immediate suction, yielding 37 L of serous fluid (Figure 4). Traction on the cyst wall edges with clamp forceps prevented spillage, and the opening was closed with 0-Prolene sutures. Attention was then turned to definitive resection for which a left salpingo-oophorectomy and infracolic omentectomy were performed (Figure 5). Peritoneal washings and random parietal peritoneal biopsies were obtained. The appendix was inspected and found grossly normal and, hence, was preserved. Finally, a 30 Fr drain was placed in the pouch of Douglas before abdominal closure. The abdomen was closed in layers. Minimal free fluid was noted in the pouch of Douglas.

Gross examination revealed a 37.5 × 28 × 10.6 cm multiloculated cystic tumor of the left ovary. Microscopy showed gastric-type mucinous epithelium with borderline (atypical proliferative) features, foci of intraepithelial carcinoma, and microinvasion (largest <5 mm), International Federation of Gynecology and Obstetrics 1A stage. A sarcoma-like spindle-cell nodule with osteoclast-like giant cells and mixed inflammation was present. An incidental 0.5 cm benign Brenner tumor was identified in the ovarian stroma. Immunohistochemistry demonstrated positivity for CK7, CK20, CEA, and CA 19-9, and negativity for CDX2, SATB2, CA 125, ER, PR, p16, vimentin, WT1, PAX8, and napsin A. Cytology and peritoneal biopsies were negative for invasive disease. Histopathological examination of the omental specimen was negative for invasive disease. There was no histological evidence of microinvasion or capsular involvement.

The patient was transferred to the intensive care unit for 24 hours of close monitoring. She received two units of packed red blood cells and two units of fresh frozen plasma, with continuous hemodynamic surveillance and carefully titrated diuresis to prevent pulmonary edema. Aggressive pulmonary physiotherapy facilitated rapid respiratory recovery. By postoperative day two, her dyspnea had resolved, and she had returned to her baseline functional status, allowing transfer to the gynecology ward. She was discharged home in good condition on postoperative day five, having made a full recovery. For postoperative venous thromboembolism prophylaxis, subcutaneous tinzaparin 4,500 IU once daily was given for 20 days.

Following review of the final pathology report, the multidisciplinary oncology board convened to discuss long‑term management. After comprehensive counseling, the patient and her family were informed regarding risks such as familial predisposition, disease progression, and the severe psychiatric disease. The patient elected not to pursue fertility preservation. Four weeks later, she underwent an uncomplicated total abdominal hysterectomy with right salpingectomy and appendectomy. Histopathological examination of the surgical specimen confirmed the complete absence of residual disease.

Following the surgical procedure, the case was reviewed once more by the Oncological Tumor Board. It was determined that the treatment phase was completed, and follow-up of the survivor was recommended. At the six-month follow-up, she remained in good health without evidence of recurrence.

## Discussion

Early recognition and multidisciplinary planning are essential when managing giant ovarian masses complicated by respiratory compromise, as illustrated by this case of a 34-year-old woman with a mucinous borderline ovarian tumor.

Giant ovarian cysts are defined as any cystic lesion exceeding 10 cm in maximal diameter, which are now exceedingly rare due to the widespread use of routine gynecological examinations and ultrasonography. In some cases, for various reasons, when gynecologic routine examination is not performed, these cysts may overgrow and fill the entire peritoneal cavity before detection, complicating the diagnosis because they are frequently misdiagnosed as ascites [6]. Although mucinous tumors most often occur in women in the fifth and sixth decades of life, they can present in young patients and are histologically classified into benign, borderline, and malignant subtypes. Borderline mucinous tumors (atypical proliferative mucinous tumors) are further subdivided into intestinal type (approximately 85%) and seromucinous type (approximately 15%) and carry a risk of progression to invasive carcinoma if left untreated [2].

Giant ovarian tumors exert compressive effects on adjacent viscera and vascular structures, leading to symptoms such as progressive abdominal distension, early satiety, constipation, urinary frequency, and high-grade dyspnea due to diaphragmatic elevation. Rarely, massive cysts have been reported to cause bowel ischemia, hydronephrosis, cardiac failure, and deep venous thrombosis through impaired venous return [8,9]. Acute presentations may involve torsion or spontaneous rupture, precipitating an acute abdomen [8]. Imaging plays a central role in preoperative assessment. Transvaginal ultrasound demonstrates sensitivities of approximately 77% and specificities of 83% for borderline ovarian tumors, while MRI offers slightly higher sensitivity (85%) but lower specificity (74%) compared with ultrasound [10]. In practice, the absence of overt mural nodules or papillary excrescences may limit the distinction between benign, borderline, and frankly malignant lesions on both ultrasound and MRI [11].

Serum tumor markers assist in risk stratification. CA 125, CA 19-9, and human epididymis secretory protein 4 (HE4) have all been evaluated, and the Risk of Ovarian Malignancy Algorithm (ROMA) algorithm (which combines CA 125, HE4, and menopausal status) has demonstrated superior accuracy in distinguishing borderline or malignant tumors from benign cysts [12]. In this patient, markedly elevated CA 19-9 (639 U/mL) with only mildly raised CA 125 (55 U/mL) supported the diagnosis of a mucinous neoplasm.

Although the overall rate of malignancy in giant ovarian cysts exceeds 10%, the nonspecific nature of early symptoms underscores the need to consider malignancy in every large ovarian lesion. In resource-limited settings, reliance on multiple serum markers may be constrained by cost, making immunohistochemistry of excised specimens particularly valuable for definitive subtype diagnosis and guiding further management [13]. This case highlights the diagnostic and operative challenges posed by giant mucinous borderline ovarian tumors and underscores the importance of comprehensive preoperative imaging, judicious use of tumor markers, and coordinated surgical planning to optimize patient outcomes.

Intraoperative drainage of fluid eliminates the occurrence of splanchnic shock, which occurs when the constrained splanchnic vascular bed proximal to the inferior vena cava is relieved with the removal of the tumor [6,12].

*KRAS* mutations remain the most frequent driver, *TP53* alterations and *HER-2* amplifications are still uncommon, and overexpression of mucin genes (*MUC2*, *MUC5AC*, *MUC6*) continues to fuel cyst expansion. Imaging workup and tumor markers (CEA, CA-125, CA 19-9) are used in the same way. Histologically, however, malignant borderline tumors with microinvasion are distinguished by one or more small foci of stromal infiltration (<5 mm in greatest dimension), often accompanied by a subtle desmoplastic response [2,14]. This microinvasion does carry a slightly higher risk of recurrence, particularly if margins are close or if the lesion is incompletely resected, but it does not generally alter the favorable overall prognosis or the surgical management paradigm. The only addition is scrutiny for and reporting of those tiny invasive foci, as they modestly raise the risk of local recurrence [2,5,14].

Fertility-sparing surgery, typically unilateral salpingo-oophorectomy with preservation of the uterus and contralateral ovary, can be considered in young patients with stage I borderline tumors confined to one ovary, especially when there is no invasive or micropapillary component and margins are clear. An infracolic omentectomy should always be done, and in suspicious mucinous ovarian tumors, the appendix should be examined for lesions and resected if pathological implants are found [15]. However, this approach should be abandoned in the presence of bilateral disease, clear microinvasion beyond 5 mm, high‐risk histologic features (such as micropapillary architecture), elevated stage or positive peritoneal implants, or when the patient’s personal or family history suggests a strong hereditary cancer syndrome. Additionally, patient choice plays a decisive role. If a woman does not wish to preserve fertility or expresses significant anxiety about recurrence, definitive surgery is the prudent option [15].

Paracentesis in the setting of a suspected ovarian cystic mass should be strictly avoided, as blind fluid removal carries risks of cyst rupture, chemical peritonitis, tumor cell dissemination, and diagnostic delay. The American College of Obstetricians and Gynecologists Practice Bulletin No. 174 recommendations explicitly counsel against paracentesis in these cases [16]. Instead, when decompression is necessary, controlled intraoperative cyst drainage via a small cystotomy under direct visualization is recommended to preserve specimen integrity for histopathologic assessment and minimize associated complications, as reported in similar cases [17,18].

Although minimally invasive surgery offers shorter hospitalization and reduced postoperative morbidity, it should be reserved for appropriately selected borderline ovarian tumors of moderate size. In a retrospective series, Maneo et al. found that laparoscopic treatment of borderline tumors yielded higher rates of cyst rupture and persistent disease when the mass exceeded 5 cm, leading them to recommend reserving laparoscopy for lesions ≤5 cm [19]. Similarly, Ødegaard et al. reported that laparoscopy for stage I borderline tumors under 10 cm resulted in fewer complications and shorter hospital stays compared with laparotomy, but that intraoperative rupture risk rose significantly for masses >10 cm [20]. In our opinion, laparotomy remains the gold standard for giant ovarian cysts (>12 cm) to ensure intact specimen removal, allow comprehensive surgical staging, and minimize the risk of peritoneal spillage.

## Conclusions

Managing giant ovarian tumors demands a multidisciplinary, patient-centered strategy that begins with high-resolution ultrasound and MRI to accurately delineate tumor size, complexity, and potential high-risk features. CT should be reserved for urgent conditions in which MRI is not available or for staging malignant cases. Intraoperatively, controlled decompression, such as a small cystotomy under direct vision with immediate suction, helps prevent sudden hemodynamic shifts and peritoneal spillage, while careful histopathologic assessment of the cyst wall and contents ensures any microinvasion or adverse histologic patterns are not overlooked. Postoperative surveillance tailored to each patient’s risk profile, combining periodic imaging (ultrasound or MRI) with serum markers (CA-125, CA 19-9, HE4), which enables early detection of the rare recurrence. Equally important is providing clear counseling on fertility preservation and definitive surgery according to the guidelines by always prioritizing the oncological safety of the patient.
